# Structural characterizations of the chloroplast translocon protein Tic110

**DOI:** 10.1111/tpj.12249

**Published:** 2013-05-25

**Authors:** Jia-Yin Tsai, Chiung-Chih Chu, Yi-Hung Yeh, Lih-Jen Chen, Hsou-min Li, Chwan-Deng Hsiao

**Affiliations:** Institute of Molecular Biology, Academia SinicaTaipei, 11529, Taiwan

**Keywords:** *Cyanidioschyzon merolae*, *CMQ342C*, chloroplast, translocon, Tic110, HEAT repeats

## Abstract

Tic110 is a major component of the chloroplast protein import translocon. Two functions with mutually exclusive structures have been proposed for Tic110: a protein-conducting channel with six transmembrane domains and a scaffold with two N-terminal transmembrane domains followed by a large soluble domain for binding transit peptides and other stromal translocon components. To investigate the structure of Tic110, Tic110 from *Cyanidioschyzon merolae* (CmTic110) was characterized. We constructed three fragments, CmTic110_A_, CmTic110_B_ and CmTic110_C_, with increasing N-terminal truncations, to perform small-angle X-ray scattering (SAXS) and X-ray crystallography analyses and Dali structural comparison. Here we report the molecular envelope of CmTic110_B_ and CmTic110_C_ determined by SAXS, and the crystal structure of CmTic110_C_ at 4.2 Å. Our data indicate that the C-terminal half of CmTic110 possesses a rod-shaped helix-repeat structure that is too flattened and elongated to be a channel. The structure is most similar to the HEAT-repeat motif that functions as scaffolds for protein–protein interactions.

## Introduction

Chloroplasts are essential organelles in plants and perform many important functions such as photosynthesis, amino acid synthesis and nitrogen assimilation. Most chloroplast proteins are nuclear encoded, synthesized as precursor proteins with an N-terminal transit peptide, and imported into chloroplasts via the translocon complex located at the two envelope membranes of chloroplasts. Translocon components in the outer and inner envelope membranes are called Toc and Tic (translocon at the outer- and inner-envelope membrane of chloroplasts) proteins, respectively. Toc75, Toc34 and Toc159 are the major Toc components. Toc34 and Toc159 are membrane-associated GTPases acting as receptors for precursors. Toc75 is a β-barrel membrane protein and functions as the protein-conducting channel across the outer membrane. Many Tic proteins have been identified. They are proposed to function in precursor protein transport across the intermembrane space, in inner-membrane channel formation or in redox regulations (for reviews, see Jarvis, 2008; Kessler and Schnell, 2009; Kovacs-Bogdan *et al*., 2010; Li and Chiu, 2010). Three stromal ATPases, Hsp90C (Inoue *et al*., 2013), cpHsc70 (Shi and Theg, 2010; Su and Li, 2010), and Hsp93 (ClpC; Constan *et al*., 2004; Nielsen *et al*., 1997) are shown to be important for driving precursor protein translocation into the stroma.

Three proteins, Tic20, Tic21 and Tic110, have been proposed to function as protein-conducting channels across the inner envelope membrane. Tic20 and Tic21 are both about 20 kDa in size with four transmembrane helices, similar to amino acid permeases (Kouranov *et al*., 1998; Teng *et al*., 2006). Tic20 has been shown to form a cation-selective channel by itself (Kovacs-Bogdan *et al*., 2011) and a precursor-protein-sensitive channel together with three other Tic components (Kikuchi *et al*., 2013). Tic21 has been shown genetically to function in the same pathway as Tic20 (Teng *et al*., 2006). Furthermore, a complex containing Tic20, Tic21 and translocating precursors, but not Tic110, has been identified in chloroplasts (Kikuchi *et al*., 2009, 2013).

**Figure 1:**
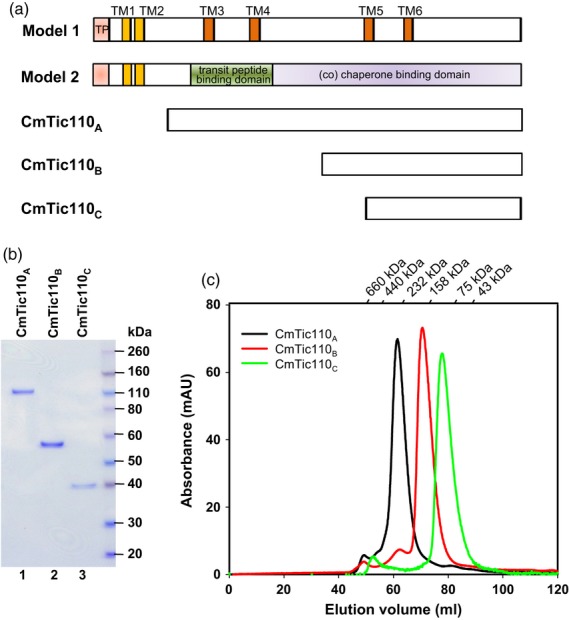
Purification of the three CmTic110 proteins. (a) Schematic representations of the two structural models of Tic110 and the corresponding regions covered by CmTic110_A_, CmTic110_B_ and CmTic110_C_. Locations of the proposed transmembrane domains (TM1 and TM2 are represented by yellow boxes; TM3 to TM6 are represented by orange boxes), transit peptides (TP, pink boxes) and domains for transit-peptide binding (green box) and (co) chaperone binding (purple box) are marked. (b) CmTic110 proteins were purified to homogeneity. One microgram of purified proteins was analyzed by SDS-PAGE and stained by Coomassie blue. (c) Purified CmTic110 proteins were analyzed by gel filtration. The chromatograph of each CmTic110 protein is shown.

Tic110 is an essential translocon component (Inaba *et al*., 2005; Kovacheva *et al*., 2005). Two models have been presented for its topology and function. The first model describes Tic110 with six transmembrane helices (herein referred to as TM1 to TM6; Figure 1a, model 1) located throughout the polypeptide. The first two transmembrane helices (TM1 and TM2) function as a signal-anchor sequence to target the protein to the inner membrane (Lübeck *et al*., 1997), and the rest of the polypeptide traverses the inner membrane four more times (TM3 to TM6) to form a Ca^2+^-sensitive and cation-selective channel (Heins *et al*., 2002; Balsera *et al*., 2009; Kovacs-Bogdan *et al*., 2011). Tic110 is therefore proposed to be the major channel for translocation of protein across the inner membrane. The second model also shows Tic110 with a membrane anchor composed of TM1 and TM2, but the rest of the protein is entirely soluble and localized in the stroma (Figure 1a, model 2; Inaba *et al*., 2005, 2003; Jackson *et al*., 1998). The N-terminal portion of the soluble domain binds transit peptides directly (Inaba *et al*., 2003). Tic110 has also been shown to interact with the stromal chaperones Hsp60, Hsp93 and Hsp90C and the stromal domain of Tic40 (Kessler and Blobel, 1996; Kouranov *et al*., 1998; Chou *et al*., 2006; Chu and Li, 2012; Inoue *et al*., 2013). Tic110 is thus proposed to function as a scaffold in the stroma for binding transit peptides emerging from the inner-membrane channel, and for recruiting/tethering other translocon components located in the stroma.

Structurally the two models for Tic110 are mutually exclusive. To investigate the structure of Tic110, we cloned and expressed the Tic110 homologue from *Cyanidioschyzon merolae* 10D, herein named CmTic110, for structural studies. *Cyanidioschyzon merolae* is a model red alga living in acidic hot springs, and its genome has been sequenced (Matsuzaki *et al*., 2004). Small-angle X-ray scattering (SAXS) analyses of recombinant CmTic110 fragments revealed an elongated shape in solution. Crystallography combined with Dali searches shows a HEAT-repeat structure for a C-terminal fragment of CmTic110. These data indicate that the C-terminal part of Tic110 possesses the structural characteristics of a scaffold for protein–protein interactions.

## Results

### Sequence analyses of Tic110 from *Cyanidioschyzon merolae*

**Figure 2:**
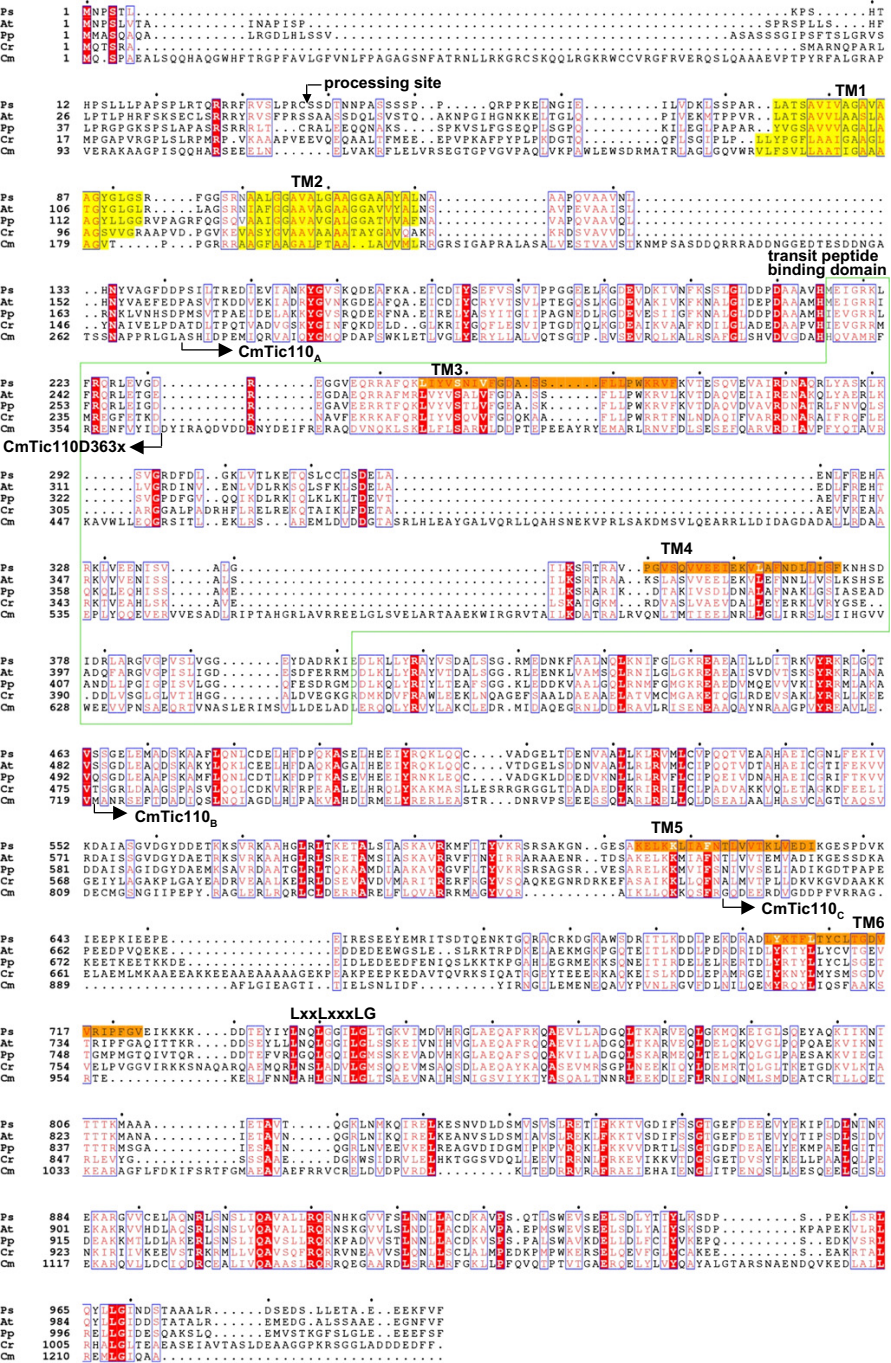
Sequence alignment and domain features of Tic110s. Tic110 homologues from *Pisum sativum* (Ps), *Arabidopsis thaliana* (At), *Physcomitrella patens* (Pp), *Chlamydomonas reinhardtii* (Cr) and *Cyanidioschyzon merolae* (Cm) were aligned by clustalW2, generated from ESPript (Gouet *et al*., 1999) and further manually adjusted. Fully conserved residues are shaded in red and regions with similar residues are boxed in blue. The transit-peptide processing site in PsTic110 is indicated by a downward arrow. The transmembrane helices are shaded in yellow and orange. The region corresponding to the transit-peptide-binding domain of model 2 is boxed in green. The N termini of CmTic110_A_, CmTic110_B_ and CmTic110_C_ are marked with rightward arrows. The C terminus of CmTic110D363x is marked with a leftward arrow. The conserved leucine-zipper-like motif (L-x-x-L-x-x-x-L-G) is indicated above the sequence.

Tic110 is conserved from glaucophytes, red algae to flowering plants and is almost always encoded by a single gene, except in organisms like *Physcomitrella patens* in which the entire genome was recently duplicated (Kalanon and McFadden, 2008; Shi and Theg, 2013). We cloned and expressed Tic110 homologues from several flowering plants for crystallography studies, but without success. We then cloned and expressed the Tic110 homologue from *C. merolae*. Tic110 is encoded by a single gene (*CMQ342C*, http://merolae.biol.s.u-tokyo.ac.jp/) in the genome of *C. merolae* 10D (Matsuzaki *et al*., 2004). We compared its polypeptide sequence (CmTic110) with Tic110s from the green alga *Chlamydomonas reinhardtii* (CrTic110), the moss *P. patens* (PpTic110), and the flowering plants *Arabidopsis thaliana* (AtTic110) and pea (*Pisum sativum*, PsTic110). Transit peptide processing sites and transmembrane helices were predicted using ChloroP (Emanuelsson *et al*., 1999) and TMpred (Hofmann and Stoffel, 1993), respectively. As shown in Figure 2, CmTic110 is predicted to have a chloroplast-targeting transit peptide at its N terminus, followed by the two transmembrane helices agreed upon by both models (TM1 and TM2, Figure 2; shaded in yellow), and a large C-terminal region, similar to other Tic110s. As previously reported (Balsera *et al*., 2009), CmTic110 has similar secondary structure features to Tic110s from green plants except that it contains several insertions in the regions between TM2 and TM3, and between TM3 and TM4 of model 1. The C-terminal half contains a leucine-zipper-like motif (L-x-x-L-x-x-x-L-G; Figure 2) conserved in all Tic110s (Kalanon and McFadden, 2008). Secondary structure prediction by PHYRE2 (Kelley and Sternberg, 2009) also shows that CmTic110 exhibits an all α-helix structure (green tubes in Figure S1) like other Tic110s. This result also agrees with circular dichroism spectra of Tic110 from pea and Arabidopsis, both of which show a high α-helix content (Inaba *et al*., 2003; Balsera *et al*., 2009).

### CmTic110 is a membrane protein in chloroplasts

**Figure 3:**
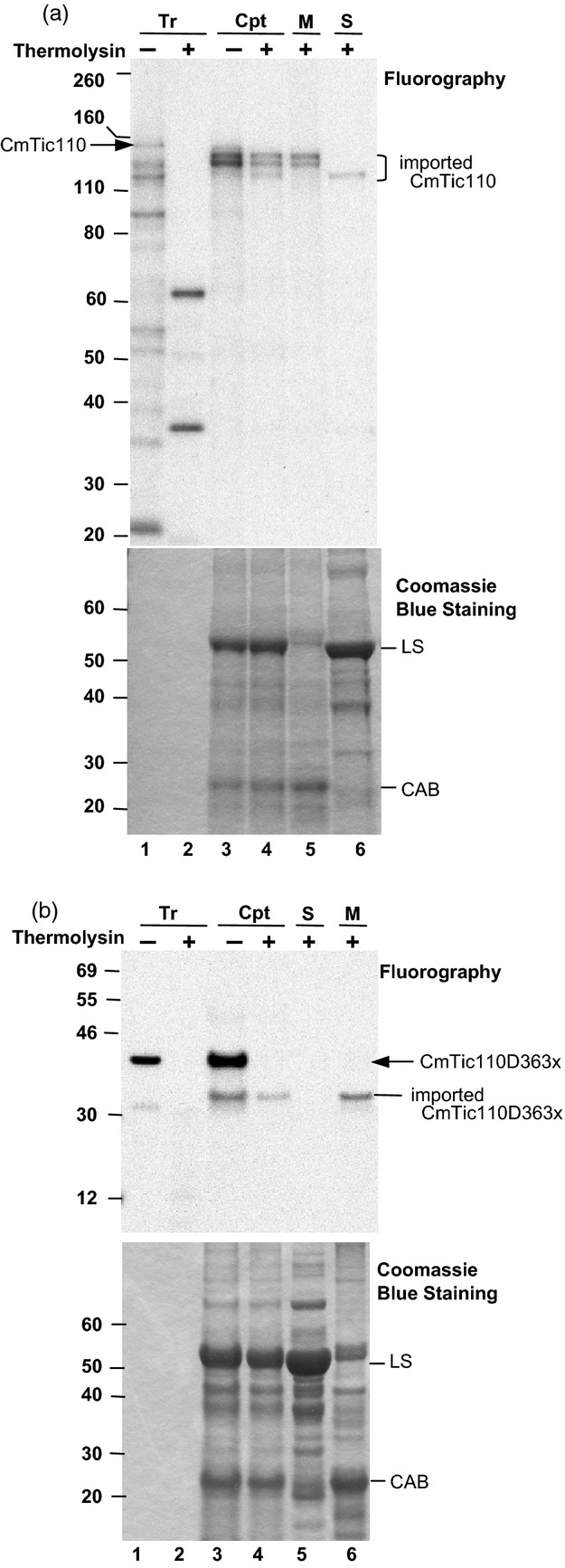
CmTic110 is imported into pea chloroplasts. (a) *In vitro* translated [^35^S]Met-CmTic110 (Tr, lane 1) was treated with thermolysin directly (lane 2) or incubated with isolated pea chloroplasts (Cpt, 100 μg chlorophyll) under import conditions for 30 min. After import, a small portion of the chloroplasts (10 μg chlorophyll) were centrifuged through a 40% Percoll cushion to re-isolate intact chloroplasts (lane 3). The rest of the chloroplasts were digested with thermolysin, and then recovered through a 40% Percoll cushion (lane 4). Some of the thermolysin-treated chloroplasts (81 μg chlorophyll) were further lysed hypotonically and separated into membrane (M, lane 5) and soluble (S, lane 6) fractions by centrifugation. Samples were analyzed by SDS-PAGE, Coomassie blue staining and fluorography. Twenty micrograms of proteins were loaded in lanes 3–6. The arrow indicates the full-length CmTic110 and the bracket marks the three imported and process CmTic110 inside chloroplasts. LS, large subunit of ribulose biphosphate carboxylase in the stroma, which serves as a marker for the soluble fraction; CAB, chlorophyll *a*/*b*-binding protein of the thylakoid membrane, which serves as a marker for the membrane fraction. (b) Import of CmTic110D363x. The experimental conditions and lane designations are the same as (a), except lane 5 is the soluble fraction and lane 6 is the membrane fraction.

To confirm that CmTic110 possesses a transit peptide for chloroplast targeting, CmTic110 was synthesized by *in vitro* transcription and translation and incubated with pea chloroplasts under import conditions. The full-length CmTic110 was synthesized as an approximately 150-kDa protein (Figure 3a, lane 1, arrow). After import, it was processed to three smaller-sized proteins, all around 130–140 kDa (Figure 3a, bracket). These proteins were resistant to thermolysin treatments performed on chloroplasts after import (Figure 3a, lane 4), indicating that they were inside chloroplasts. Chloroplasts after import were further lysed hypotonically and separated into membrane and soluble fractions. The two higher-molecular-weight imported proteins were in the membrane fraction. The smallest protein was in the soluble fraction (Figure 3a, lanes 5 and 6). This protein might be a degradation product after the loss of the transmembrane helices at the N terminus. These data suggest that CmTic110 possesses a functional chloroplast-targeting transit peptide and imported CmTic110 was similar in size to higher plant Tic110s and anchored in the membrane.

Many smaller polypeptides were produced in the *in vitro* CmTic110 translation reaction (Figure 3a, lane 1). Efforts to reduce the number of these fragments were unsuccessful. To rule out the possibility that these fragments have contributed to the imported proteins observed, and to get a better estimation of the size of CmTic110 transit peptide, we constructed a C-terminally truncated CmTic110, CmTic110D363x, in which the codon encoding Asp of residue 363 was mutated to a stop codon. CmTic110D363x was synthesized as a protein of approximately 42 kDa (Figure 3b, lane 1, arrow). After import, it was processed to a mature protein of 32 kDa. The mature protein was inside chloroplasts, judging from its thermolysin resistance (Figure 3b, lane 4), and was located entirely in the membrane fraction (Figure 3b, lane 6). This result confirms that CmTic110 could be imported into chloroplasts and further showed that CmTic110 has a transit peptide of approximately 10 kDa, placing the processing site very close to the region corresponding to the processing site of pea Tic110.

### Recombinant CmTic110s without TM1 and TM2 are soluble proteins

To characterize the properties of CmTic110 in solution, we designed a series of N-terminally truncated constructs lacking TM1 and TM2 to produce recombinant proteins in *Escherichia coli*. As shown in Figures 1(a) and 2, CmTic110_A_, composed of residues 273–1218, corresponds to almost the complete region after TM2. CmTic110_B,_ composed of residues 720–1218, further lacks TM3 and TM4 of model 1. CmTic110_C_, composed of residues 871–1218, contains the corresponding region from the middle of TM5 to the C terminus. All of the constructs were successfully expressed and purified as soluble proteins from *E. coli*. The SDS-PAGE analyses indicated that they were purified to homogeneity (Figure 1b). Size exclusion chromatography indicated that all three proteins formed one major conformation in solution (Figure 1c). These data agree with published results showing that pea Tic110 without TM1 and TM2 was over-expressed as a soluble protein in *E. coli* (Balsera *et al*., 2009), and Arabidopsis Tic110 without TM1 and TM2 was over-expressed as a soluble protein in both *E. coli* and Arabidopsis chloroplasts (Inaba *et al*., 2003, 2005).

### Small-angle X-ray scattering analyses of CmTic110s

**Figure 4:**
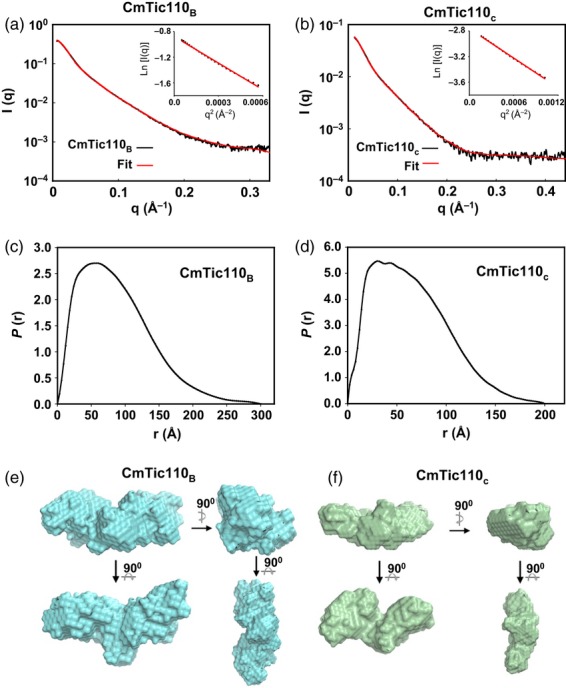
Small-angle X-ray scattering (SAXS) characterizations of CmTic110_B_ and CmTic110_C_. (a, b) Experimental X-ray scattering curves (black line) and the theoretical fitting curves (red line) of CmTic110_B_ and CmTic110_C_ were generated with gnom. The insets show the Guinier plots. (c, d) The distance distributions of CmTic110_B_ and CmTic110_C_. (e,f) Averaged low-resolution envelope obtained from 10 independent *ab initio* models of CmTic110_B_ and CmTic110_C_ derived from the SAXS data.

Since all the recombinant CmTic110 proteins were in soluble form, we first employed SAXS to determine their molecular shape in solution. Data were obtained for CmTic110_B_ and CmTic110_C_ but not for CmTic110_A_ because CmTic110_A_ was prone to aggregation. Values for the radius of gyration (*R*_g_) for CmTic110_B_ and CmTic110_C_ obtained from a Guinier plot using the primus program are 66.5 and 49.2 Å, respectively (Table S1). The SAXS data and the gnom curve fitting are shown in Figure 4(a,b). The maximum dimension (*D*_max_), determined to be about 300 Å for CmTic110_B_ and 200 Å for CmTic110_C_, was generated from the distance distribution [*P*(*r*)] functions (Figure 4c,d). Based on the scattering data, 10 independent models were generated using the program gasbor without any symmetry constraint. These 10 models were averaged by damever. Surface envelopes calculated from the data showed that both CmTic110_B_ and CmTic110_C_ have an elongated zigzag shape (Figure 4e,f). The structure of CmTic110_C_ can be fitted into the central portion of CmTic110_B_ (Figure S2c), suggesting that the structure of CmTic110_B_ is an extension of that of CmTic110_C_. In addition, the hydrodynamic radius (*R*_h_) of CmTic110_B_ and CmTic110_C_ particles, determined by dynamic light scattering, was calculated to be 73 and 50 Å, respectively (Figure S3), consistent with their *R*_g_ obtained from SAXS.

### Crystal structure of CmTic110_C_

**Figure 5:**
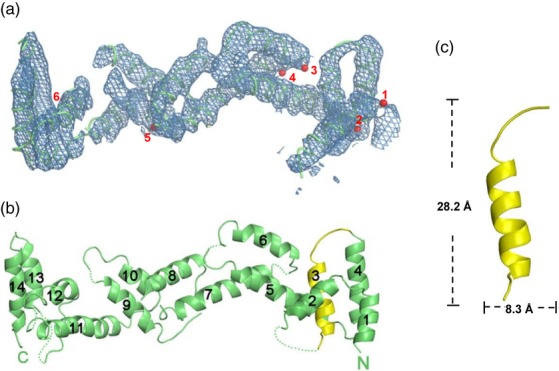
Structure of CmTic110_C_. (a) Electron density map of experimental phase at 1.5σ and model building of CmTic110_C_ based on the six selenium sites (red balls 1–6 for selenium in Met917, Met940, Met1017, Met1020, Met1051 and Met1212, respectively). (b) A ribbon drawing of the CmTic110_C_ structure. Helix 3 and part of the loop connecting helix 4 (indicated in yellow) is the corresponding region of TM6 in model 1 shown in Figure 1(a). (c) A close-up view of the region corresponding to TM6 and its dimensions. All the figures were generated with pymol (http://www.pymol.org/).

We next subjected the three CmTic110 proteins to crystallization trials; however, crystals suitable for X-ray diffraction were only obtained from CmTic110_C_. The crystal structure of CmTic110_C_ was determined by selenium single-wavelength anomalous dispersion (Se-SAD) at a resolution of 4.2 Å. The electron density map and overall structure tracing are depicted in Figure 5(a). Our structure covers residues 903–1213, missing the first 32 and the last five residues of the CmTic110_C_ construct. The structure comprises 14 α-helices of seven continuous anti-parallel helix-loop-helix pairs (Figure 5b), forming an elongated shape of about 96 × 28 × 15 Å. Several loops linking the helices were not located (dashed lines in Figure 5b). Due to the low resolution, CmTic110_C_ is assigned as a poly-Ala structure, except for the six Met residues. According to model 1 of the Tic110 structure (Figure 1a), the construct of CmTic110_C_ contains TM6 and part of TM5. The region corresponding to TM5 could not be observed in our structure due to high flexibility in the N-terminal end. The region corresponding to TM6 consists of helix 3 and part of the loop connecting helix 4 in our structure (Figure 5b,c, colored in yellow). This region is 28.2 Å high, much smaller than the 60–80 Å thickness observed for the chloroplast inner envelope membrane (Bisalputra and Bailey, 1973; Heber and Heldt, 1981). Therefore, this helix is unlikely to cross the inner membrane.

To search for possible functions of the CmTic110_C_ folding, we carried out a structure-based homology analysis by Dali (Holm and Rosenstrom, 2010) with the Protein Data Bank. No membrane protein with a similar folding was found. From the Dali results, the protein Sra1 in the WAVE regulatory complex (3p8c) (Chen *et al*., 2010), was identified as the closest structural homologue with a root mean square deviation (rmsd) of 4.3 Å over 145 residues (Cα atoms, Figure S4a). Other structural homologues include the cullin Cul4A in the damage-specific DNA-binding protein 1 (DDB1)-Cul4A-Roc1-SV5-V complex (2hye; rmsd of 4.0 Å over 148 residues; Figure S4b) (Angers *et al*., 2006), nuclear pore complex protein Nup107 (3i4r; rmsd of 2.6 Å over 70 residues; Figure S4c) (Whittle and Schwartz, 2009) and the cullin Cul1 in the Cul1-Rbx1-Skp1-F box^Skp2^ complex (1ldj; rmsd of 3.1 Å over 77 residues; Figure S4d) (Zheng *et al*., 2002). These proteins all show an elongated shape formed by helical repeats that can be classified as the HEAT-like repeat structure. The structure is usually used to form complexes with other proteins. The presence of HEAT-like repeat structure in CmTic110_C_ also agrees with a previous report indicating that pea Tic110 has several predicted HEAT repeats (Balsera *et al*., 2009). However, the report only indicated six HEAT repeats located in the regions between TM2 and TM3, between TM4 and TM5 and in the C terminus after TM6. We thus reanalyzed pea Tic110 and CmTic110 using hhrepid (Biegert and Soding, 2008), a repeated-protein-motif prediction server specifically designed for HEAT repeats. Because the program only allows 800 amino acids per entry, the last 800 amino acids of pea Tic110 (residues 197–996) and CmTic110 (residues 419–1218) were analyzed. The results showed that pea Tic110 was predicted to have eight HEAT repeats located throughout the polypeptide (Figure S5) and CmTic110 was predicted to have 12 HEAT repeats (Figure S1, magenta tubes, and Figure S5). In the region resolved by our crystal structure in CmTic110_C_, HHrepID predicted four HEAT repeats located in helices 4–5, 6–7, 8–9 and 10–11. Our structure indicates that helices 5–6, 7–8, 9–10 and 11–12 form four HEAT repeats. Even though the repetitive helix pairs are shifted by one helix, the prediction of the HEAT repeat structure is accurate.

It has been shown that recombinant pea Tic110 forms dimers in solutions. It has a calculated molecular mass around 100 kDa, but its retention volume in gel filtration experiments corresponded to a globular protein of 400 kDa. It was suggested that the higher than calculated molecular mass was due to a possible non-globular shape of Tic110 (Balsera *et al*., 2009). Pea and Arabidopsis Tic110 also migrated as dimers on blue native (BN)-PAGE when solubilized from chloroplast membranes (Kikuchi *et al*., 2009, 2013). The calculated molecular masses of CmTic110_B_ and CmTic110_C_ are 59.9 and 42.8 kDa, respectively. The molecular masses for CmTic110_B_ and CmTic110_C_ estimated from our SAXS data were 172 and 79.6 kDa, respectively (Table S1), in agreement with our gel filtration data (Figure 1c). Thus it is most likely that both CmTic110_B_ and CmTic110_C_ also existed as dimers in solution. The CmTic110_B_ dimer has a larger than calculated molecular mass, most likely due to its elongated shape. In the crystal packing, CmTic110_C_ formed an extended superstructure with tail-to-tail and head-to-head packing (Figure S2a). We fitted both the head-to-head and the tail-to-tail dimers with the CmTic110_C_ SAXS data using crysol (Figure S2b). The tail-to-tail dimer fits slightly better (χ-value 6.412) than the head-to-head dimer (χ-value 8.308). The chi-squared values are relatively high for both dimers, most likely because our crystal structure is a poly-Ala structure with no side chains. These data suggest that the SAXS envelopes we observed were formed by CmTic110_B_ dimers and CmTic110_C_ dimers (Figure S2c), and CmTic110_B_ and CmTic110_C_ have a similar oligomerization state to pea and Arabidopsis Tic110 in solutions.

## Discussion

All secondary structure predictions suggest that all Tic110s analyzed so far have similar structures. The biochemical properties of CmTic110 fragments are also similar to those shown for Arabidopsis and pea Tic110 (Inaba *et al*., 2003; Balsera *et al*., 2009). Although sequence comparison shows that CmTic110 has several insertions right after TM2 when compared with Tic110s from other species, these insertions are outside the CmTic110_B_ and CmTic110_C_ regions we analyzed. Thus it is most likely that Tic110s in higher plants also have a similar structure to CmTic110 in the regions we analyzed.

Our SAXS results show that CmTic110_B_ has an elongated and flattened conformation in solution. The crystal structure of CmTic110_C_ further shows an elongated helical-repeat structure. The dimensions of CmTic110_C_ are about 96 × 28 × 15 Å. Measurements of the chloroplast inner envelope membrane from various species have estimated that the thickness of the inner membrane is about 60–80 Å (Bisalputra and Bailey, 1973; Heber and Heldt, 1981). The only way that CmTic110_C_ can cross the membrane is through the 96 Å dimension, which will bury about six hydrophilic α-helices in the membrane and is energetically extremely unlikely. However, in liposome floatation experiments, the region corresponding to our CmTic110_B_ from pea Tic110 was able to bind to liposome (the M2 fragment in Balsera *et al*., 2009). Because our resolution does not allow assignment of side chains, it is possible that this region may have some small patches of hydrophobic surfaces that allow attachment to the neutral head group of phosphatidylcholine used for the liposome experiments. Phosphatidylcholine is not present in the chloroplast inner envelope membrane (Douce and Joyard, 1990). Whether the M2 fragment will insert into liposomes with lipid compositions of the chloroplast inner envelope membrane remains to be tested. When expressed in the chloroplast stroma, the entire region after TM2 of Arabidopsis Tic110 exists as a soluble protein in the chloroplast stroma and did not insert into the inner membrane (Inaba *et al*., 2003, 2005).

In work showing that Tic110 has channel activities *in vitro*, it was shown that the first three transmembrane domains in model 1 (TM1 to TM3) did not possess channel activity when reconstituted into liposome. The channel activity was provided by the last four transmembrane domains (TM3 to TM6) (Balsera *et al*., 2009; Kovacs-Bogdan *et al*., 2010). Our structural data indicate that TM5 and TM6 are unlikely to exist. We cannot exclude the possibility that the region not covered by our analyses, the TM3 and TM4 regions of model 1, provided the channel activity observed in the reconstituted liposome. Of note is the fact that the six-transmembrane-domain topology of Tic110 was deduced using isolated inner membrane vesicles (Balsera *et al*., 2009), while experiments using intact chloroplasts have shown that the region after TM2 is entirely inside the stroma (Kessler and Blobel, 1996; Jackson *et al*., 1998; Chou *et al*., 2003; Inaba *et al*., 2003, 2005; Kikuchi *et al*., 2009). Isolated inner membrane vesicles may have mixed orientations and may complicate the experimental results.

Although the structure of the complete region after TM2 is currently unavailable, the structures of CmTic110_B_ and CmTic110_C_ provide direct evidence showing that half of the region after TM2 has a HEAT-repeat structure with an elongated shape. All structural homologues we obtained from Dali also have elongated shapes, with the HEAT repeats forming the region for interacting with partner proteins in the same complex. For example, the CmTic110_C_ structure aligns best to subdomain 4 within domain I of Sra1. This part of Sra1 is composed entirely of HEAT repeats and provides the surface for interacting with Nap1 and WAVE1 of the WAVE regulatory complex (Chen *et al*., 2010). In another example, the N-terminal long stalk-like domain of Cul1 and Cul4A, both consisting of three HEAT-like repeats and the first few repeats, which aligned best to CmTic110_C_, binds to the adapter protein Skp1 in Cul1 (Zheng *et al*., 2002) and to DDB1 in Cul4A (Angers *et al*., 2006), respectively. Therefore, the shape and structure of the Tic110 C-terminal region most likely also enable Tic110 to function as a scaffold for interacting with other proteins in the chloroplast translocon complex.

It was argued that Tic20 could not be the major protein translocation channel across the inner membrane because it was present at <1/20 of the molar ratio of Tic110 (Kovacs-Bogdan *et al*., 2011) and <1/10 that of Toc75 (Vojta *et al*., 2004). However, a recent direct analysis has shown that the stoichiometry of Tic20 to Toc75 is actually about 1:2.5 (Kikuchi *et al*., 2013). Furthermore, Tic20, together with three other essential Tic proteins, Tic56, Tic100 and Tic214, forms a precursor-sensitive channel when reconstituted in planar lipid bilayers. In chloroplasts, these four proteins form a stable 1-MDa complex, and Tic110 and Tic40 are not in this complex (Kikuchi *et al*., 2013). It is therefore most likely that the 1-MDa Tic20 complex forms the channel traversing the inner membrane, and Tic110, through binding transit peptides and scaffolding the various stromal chaperone motors and co-chaperone, functions in the next stage of the import process facilitating complete precursor translocation into the stroma.

## Experimental Procedures

### Plant materials and growth conditions

For growing pea seedlings (*P*. *sativum* cv. Little Marvel, De Bruyn Seed Co., http://www.debruynseed.com/), the imbibed seeds were grown on vermiculite for 8–10 days under a 12-h photoperiod at 20°C with a light intensity of approximately 150 μmol m^−2^ sec^−1^.

### Plasmid construction

Because the gene encoding CmTic110 contains no introns (Matsuzaki *et al*., 2004), the coding region of CmTic110 was amplified from the genomic DNA of *C. merolae* 10D by PCR using the CmTic110-F1 and CmTic110-R1 primer pair (Table S2) and cloned into the pGEM-T vector (Promega, http://worldwide.promega.com/). The sequence was confirmed by sequencing and the plasmid was named pGEM-T-CmTic110. The plasmid was then used as a template to generate the three N-terminal truncation mutants by PCR using the following primers: CmTic110_A_-F1 and CmTic110R2 for CmTic110_A_ (residues 273–1218); CmTic110_B_-F1 and CmTic110R2 for CmTic110_B_ (residues 720–1218); CmTic110_C_-F1 and CmTic110R2 for CmTic110_C_ (residues 871–1218). A *Nde*I site and a *Xho*I site were added to the forward and the reverse primers, respectively (Table S2). The fragments were digested and cloned in the *Nde*I/*Xho*I site of pET28a and the resulting plasmids were named pET28a-CmTic110_A_, pET28a-CmTic110_B_ and pET28a-CmTic110_C_, respectively. The recombinant proteins produced have a His_6_ tag at both ends. CmTic110D363x was generated from pGEM-T-CmTic110 using the QuikChange II Site-Directed Mutagenesis Kit (Agilent Technologies, http://www.chem.agilent.com/store/Default.aspx) with primers CmTic110-D363amber-F and CmTic110-D363amber-R (Table S2). The fragment corresponding to CmTic110D363x was further amplified with primers CmTic110-F5-*Hin*dIII and CmTic110-R5-*Xba*I and subcloned into the *Hin*dIII/*Xho*I site of pSP72 (Promega) to generate pSP72-CmTic110D363x.

### *In vitro* translation, protein import and post-import analyses

[^35^S]Met-CmTic110 and CmTic110D363x were *in vitro* transcribed/translated in the TNT reticulocyte lysate system (Promega). Chloroplasts were isolated from pea seedlings as described (Perry *et al*., 1991). Isolated chloroplasts were adjusted to 1 mg chlorophyll ml^−1^ in import buffer (330 mm sorbitol, 50 mm HEPES-KOH, pH 8.0) for import assays. [^35^S]Met-labeled precursor proteins were incubated with isolated chloroplasts in the presence of 3 mm ATP in import buffer at 25°C for 30 min. After import, intact chloroplasts were re-isolated through a 40% Percoll cushion at 4°C, washed once with import buffer and dissolved in protein extraction buffer containing 300 mm 2-amino-2-(hydroxymethyl)-1,3-propanediol (TRIS)-HCl, pH 8.5, 1 mm EDTA, 8% SDS (w/v) and 1 mm phenylmethylsulfonyl fluoride. Thermolysin treatment of chloroplasts after import was performed as described (Perry *et al*., 1991). To separate chloroplasts into soluble and membrane fractions, chloroplasts after import were lysed by a hypotonic buffer (25 mm HEPES-KOH, pH 7.5, 5 mm MgCl_2_) at a chloroplast concentration of 0.5 mg chlorophyll ml^−1^ and then separated into soluble and membrane fractions by ultracentrifugation at 100 000 ***g*** for 45 min at 4°C. Soluble fractions were precipitated by 10% (w/v) trichloroacetic acid, washed with 100% ice-cold acetone and dissolved in protein extraction buffer. Membrane fractions were washed with the hypotonic buffer once, collected by another ultracentrifugation at 100 000 ***g*** for 15 min at 4°C and the pellets were resuspended by the protein extraction buffer. Samples were analyzed by SDS-PAGE, Coomassie blue staining and fluorography.

### Protein expression, purification and characterizations

For protein preparations, pET28a-CmTic110_A_, pET28a-CmTic110_B_ and pET28a-CmTic110_C_ were transformed into the *E. coli* strain BL21(DE3). Protein expression was induced with 1 mm isopropyl β-d-1-thiogalactopyranoside after the OD_600_ of the culture reached about 0.6. The cells were further cultured at 37°C for another 3 h. Bacterial cells were harvested by centrifugation at 5000 ***g*** for 30 min at 4°C, resuspended in Ni-NTA column binding buffer (20 mm TRIS-HCl, pH 7.8, 500 mm NaCl and 5 mm imidazole) and lysed under high pressure using a microfluidizer. The clear cell lysates after centrifugation were purified using Ni-NTA affinity chromatography (GE Healthcare, http://www3.gehealthcare.com/en). Proteins were eluted using step imidazole concentrations. The majority of these three proteins were eluted at 200 mm imidazole. Gel filtration experiments were performed on HiLoad 16/60 SuperdexTM 200 preparation-grade columns connected to an AKTA-FPLC system (GE Healthcare) in 20 mm TRIS pH 7.8, 500 mm NaCl and 200 mm imidazole. For the preparation of the selenomethionyl derivative, pET28a-CmTic110_C_ was further transformed into BL21 (B834) for SeMet-CmTic110_C_ over-expression. The purification procedures were the same as described above.

### Small-angle X-ray scattering analyses

Protein samples for SAXS analyses were collected from gel filtration (Figure 1c) and the concentrations of proteins were determined by the Bradford dye-binding method. The SAXS experiments were performed at the Beamline 23A at National Synchrotron Radiation Research Center (NSRRC) in Taiwan (Liu *et al*., 2009) using a MARCCD165 detector. The protein sample concentration was 1 mg ml^−1^. The X-ray energy was 14.0 keV and the collection time was 300 sec. The buffer for gel filtration (20 mm TRIS pH 7.8, 500 mm NaCl and 200 mm imidazole) was used as the solvent blank. Primary data reduction was done with a NSRRC 23A homemade program. The processed data were analyzed using the atsas package (Petoukhov *et al*., 2007): the radius of gyration (*R*_g_) was calculated using primus, *P*(*r*) and *D*_max_ were calculated using gnom, low-resolution shapes of protein samples were determined *ab initio* from the scattering data by gasbor and averaged by damever, the calculated scattering curve was fitted with the structure of CmTic110_C_ using crysol, and SAXS envelopes and crystal structures are superimposed using supcomb. The molecular mass of proteins in solution determined from SAXS was done by SAXS MoW (http://www.if.sc.usp.br/∼saxs/saxsmow.html).

### Crystallization of CmTic110_C_

Crystallization trials were setup with 6 mg ml^−1^ protein using the hanging-drop vapor-diffusion method (McPherson, 1990). Initial screening was performed with a sparse-matrix screen from Hampton Research (http://hamptonresearch.com/Default.aspx). Only CmTic110_C_ crystals were obtained by a mixture of equal volumes of protein solution and reservoir buffer containing 20 mm TRIS-HCl pH 8.0, 300 mm NaCl, 18% PEG400 (v/v), 15% glycerol (v/v) and 200 mm CaCl_2_. The crystals grew to maximum dimensions of 0.1 × 0.1 × 0.2 mm within 3 days at 20°C. The selenomethionyl derivative crystals (SeMet-CmTic110_C_) were obtained under the same conditions. The crystals grew to maximum dimensions of 0.1 × 0.05 × 0.2 mm within 1 week at 20°C. However, these crystals diffracted poorly. To improve crystal quality, dehydration in a higher concentration of the original crystallization conditions with the addition of 5% tacsimate (v/v) resulted in greatly improved crystal quality. The SeMet-CmTic110_C_ crystal grown with this additive diffracted to 4.2 Å, and was used for data collection. The crystals belong to the P6_5_22 space group with unit-cell parameters *a* = *b* = 121.1 Å and *c* = 242.4 Å. A single wavelength was chosen for the data collection. A complete dataset was collected to a resolution of 4.2 Å on an ADSC Quantum-315 charge-coupled device detector using a synchrotron radiation X-ray source at Beamline BL13B1 of the NSRRC. The data were indexed and processed with hkl2000 (Otwinowski and Minor, 1997). solve (Terwilliger and Berendzen, 1999) was used to locate the Se sites and generate the initial SAD phase at a 4.5 Å resolution. The density modification was calculated with resolve (Terwilliger, 2000) using solvent flattening. XtalView (McRee, 1999) was used for examining electron density maps and manual model building. Further refinements were performed using cns (Brunger *et al*., 1998). The final model for all reflections above 2σ between 27.37 and 4.2 Å resolution was refined to 35.0% and a *R*_free_ value of 36.6% was obtained using 10% randomly distributed reflections. The crystallographic data and refinement statistics are shown in Table 1. The atomic coordinates for the CmTic110_C_ structure have been deposited in the Protein Data Bank under the accession code 4bm5.

**Table 1:** X-ray diffraction data and refinement statistics

Crystal	Se-Met derivative
Space group	P6_5_22
Wavelength (Å)	0.9787
Resolution (Å)^a^	4.2
Unit cell (Å) (*a*/*c*)	121.1/242.4
Total no. of reflections	102 418
No. of unique reflections	8171
Redundancy	12.5
Completeness (%)^a^	99.7 (99.7)
*I*/σ	27.8 (4.1)
*R*_sym_ (%)^b^	10.4 (53.2)
Refinement:	
Resolution (Å)	27.37–4.2
*R*-factor (%)	35.0
*R*_free_ (%)	36.6
Number of reflections used	6673
Number of atoms (non-hydrogen)	1351
r.m.s. deviation for bond length (Å)	0.011
r.m.s. deviation for bond angle (^o^)	2.5
Average B-factors:	
All protein atoms (Å^2^)	74.6

a Numbers in parentheses are for the highest resolution shell.

b *R*_sym_ = Σ|*Ih*−<*Ih*>|/Σ*Ih*, where <*Ih*> is the average intensity over symmetry-equivalent reflections.

## SUPPORTING INFORMATION

**Figure S1.** Secondary structure predictions of CmTic110.

**Figure S2.** Putative oligomerization of CmTic110_C_.

**Figure S3.** Dynamic light scattering measurements of CmTic110_B_ and CmTic110_C_.

**Figure S4.** Proteins with high structural homology to CmTic110_C_ identified by Dali.

**Figure S5.** HEAT repeat prediction of pea Tic110 (PsTic110) and CmTic110 using HHrepID.

**Table S1.** Data-collection and scattering-derived parameters of the small-angle X-ray scattering analyses.

**Table S2.** Sequences of primers used in this study.
