# Supplementary material for: Structural characterizations of the chloroplast translocon protein Tic110
Source: Plant J. 2013 May 25;75(5):847–57. doi: 10.1111/tpj.12249 (PMC3823011; doi:10.1111/tpj.12249)
Supplement: Supplementary file 1 [file tpj0075-0847-SD1.pdf]

Figure S1

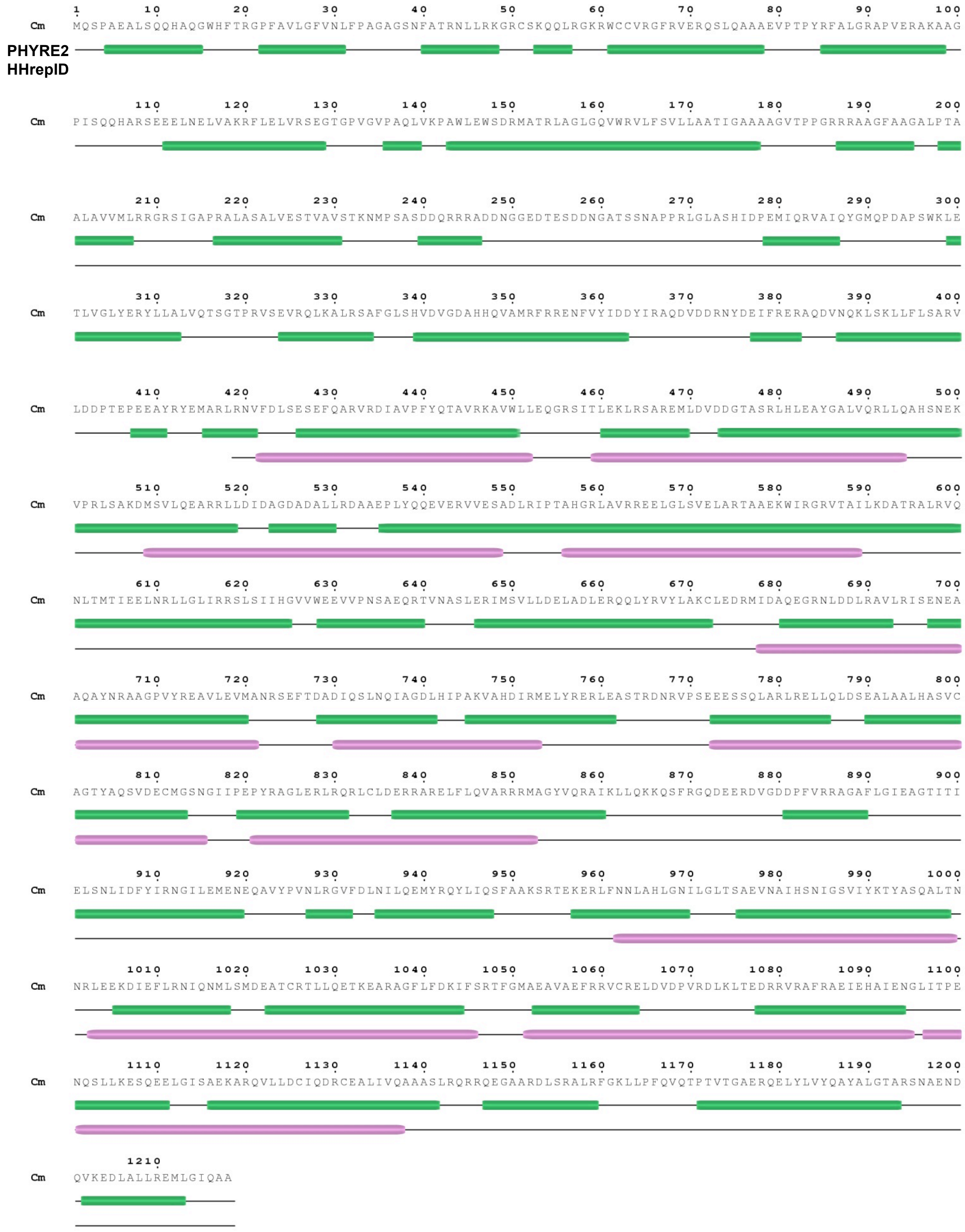
Cm
PHYRE2
HHrepID
MQSPAEALSQQHAQGWHFTRGPFAVLGFVNLFPAGAGSNFATRNLLRKGRCSKQQLRGKRWCCVRGFRVERQSLQAAAEVPTPYRFALGRAPVERAKAAG
PISQQHARSEEELNELVAKRFLELVRSEGTGPVGVPAQLVKPAWLEWSDRMATRLAGLGQVWRVLFSVLLAATIGAAAAGVTPPGRRRAAGFAAGALPTA
ALAVVMLRRGRSIGAPRALASALVESTVAVSTKNMPSASDDQRRRADDNGGEDTESDDNGATSSNAPPRLGLASHIDPEMIQRVAIQYGMQPDAPSWKLE
TLVGLYERYLLALVQTSGTPRVSEVRQLKALRSAFGLSHVDVGDAHHQVAMRFRRENFVYIDDYIRAQDVDDRNYDEIFRERAQDVNQKLSKLLFLSARV
LDDPTEPEEAYRYEMARLRNVFDLSESEFQARVRDIAVPFYQTAVRKAVWLLEQGRSITLEKLRSAREMLDVDDGTASRLHLEAYGALVQRLLQAHSNEK
VPRLSAKDMSVLQEARRLLDIDAGDADALLRDAAEPLYQQEVERVVESADLRIPTAHGRLAVRREELGLSVELARTAAEKWIRGRVTAILKDATRALRVQ
NLTMTIEELNRLLGLIRRSLSIIHGVVWEEVVPNSAEQRTVNASLERIMSVLLDELADLERQQLYRVYLAKCLEDRMIDAQEGRNLDDLRAVLRISENEA
AQAYNRAAGPVYREAVLEVMANRSEFTDADIQSLNQIAGDLHIPAKVAHDIRMELYRERLEASTRDNRVPSEEESSQLARLRELLQLDSEALAALHASVC
AGTYAQSVDECMGSNGIIPEPYRAGLERLRQRLCLDERRARELFLQVARRRMAGYVQRAIKLLQKKQSFRGQDEERDVGDDPFVRRAGAFLGIEAGTITI
ELSNLIDFYIRNGILEMENEQAVYPVNLRGVFDLNILQEMYRQYLIQSFAAKSRTEKERLFNNLAHLGNILGLTSAEVNAIHSNIGSVIYKTYASQALTN
NRLEEKDIEFLRNIQNMLSMDEATCRTLLQETKEARAGFLFDKIFSRTFGMAEAVAEFRRVCRELDVDPVRDLKLTEDRRVRAFRAEIEHAIENGLITPE
NQSLLKESQEELGISAEKARQVLLDCIQDRCEALIVQAAASLRQRRQEGAARDLSRALRFGKLLPFQVQTPTVTGAERQELYLVYQAYALGTARSNAEND
QVKEDLALLREMLGIQAA

Figure S1. Secondary structure prediction of CmTic110.

Secondary structure prediction was performed using PHYRE2. CmTic110 is predicted to have an all α-helices structure and the predicted α -helical regions are marked as green tubes under the sequence. The HEAT repeat regions predicted by HHrepID are indicated as magenta tubes below the PHYRE2 secondary structure prediction.

Figure S2

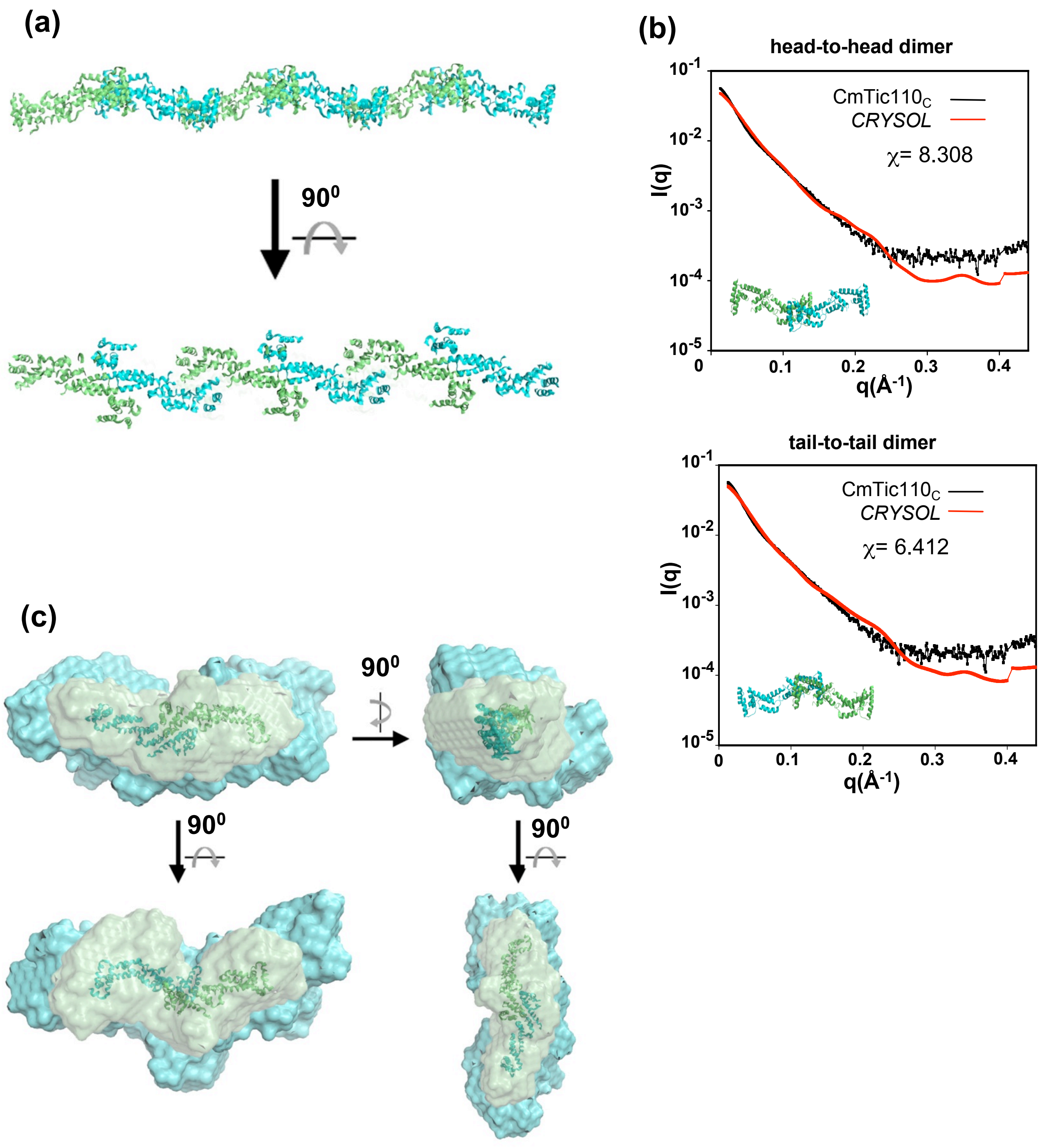

Figure S2. Putative oligomerization of CmTic110$_C$.
(a) CmTic110$_C$ extended into a super-helical structure in crystal packing. For clarity, only the C α backbone of the molecules is shown. Each monomer is colored with green or blue.
(b) The head-to-head and tail-to-tail dimers were fitted with the scattering data with CRYSOL with the χ value shown. The structures are shown in cartoon with A subunit in green and B subunit in cyan.
(b) Combining the SAXS envelopes of CmTic110$_B$ (cyan) and CmTic110$_C$ (green) and the crystal structure of the tail-to-tail dimer of CmTic110$_C$. The envelopes and structure are superimposed using the program SUPCOMB.

Figure S3

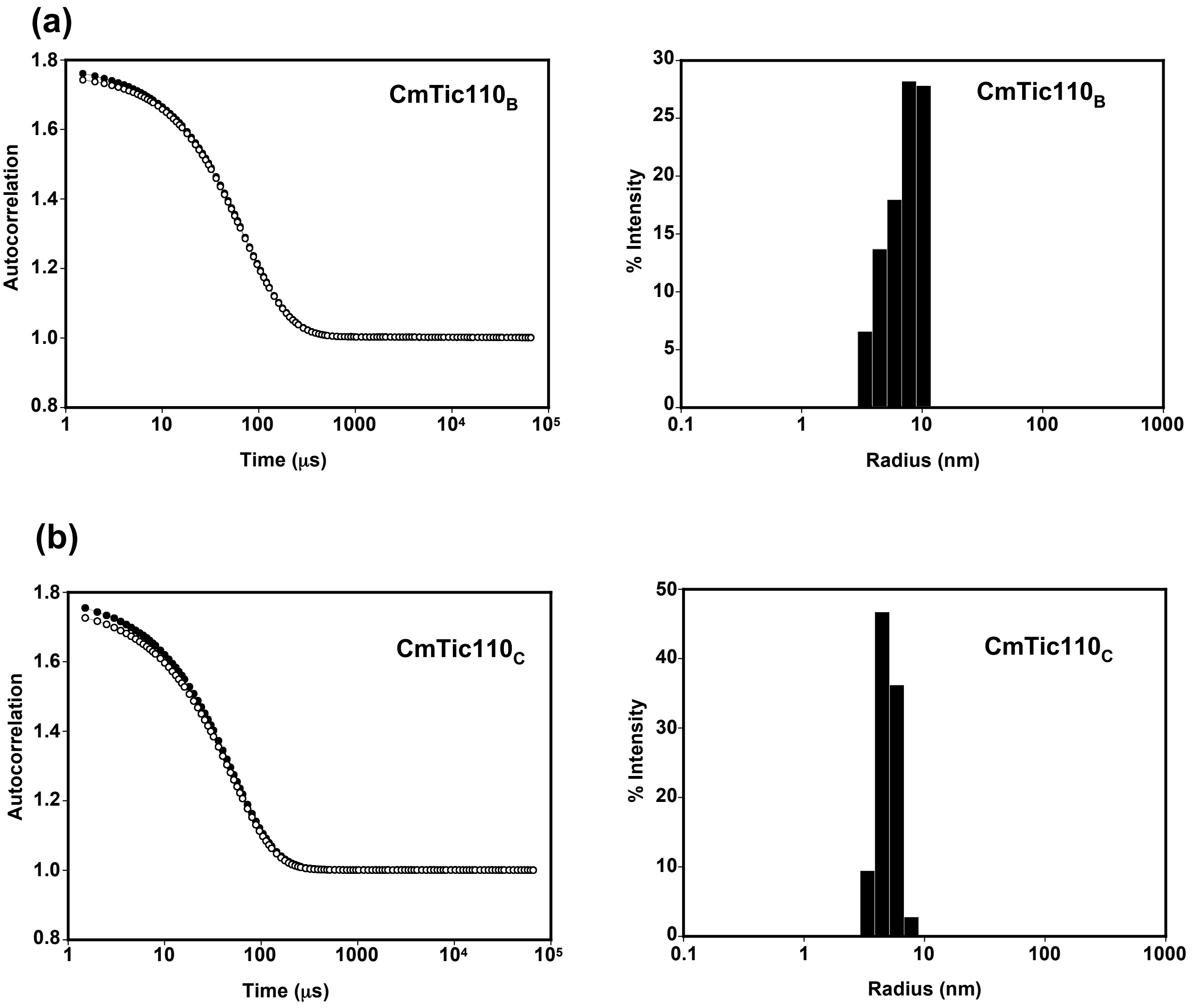

Figure S3. Dynamic light scattering measurements of CmTic110$_B$ and CmTic110$_C$. (a) and (b) Left panels show the intensity autocorrelation function of CmTic110$_B$ and CmTic110$_C$, respectively, measured by dynamic light scattering (filled circles) and the data fitting by the cumulant expansion (open circles). Size distributions of particles extracted from the autocorrelation function of both proteins are shown in the right panels. The calculated Stokes radius is 73 Å for CmTic110$_B$ and 50 Å for CmTic110$_C$.

Figure S4

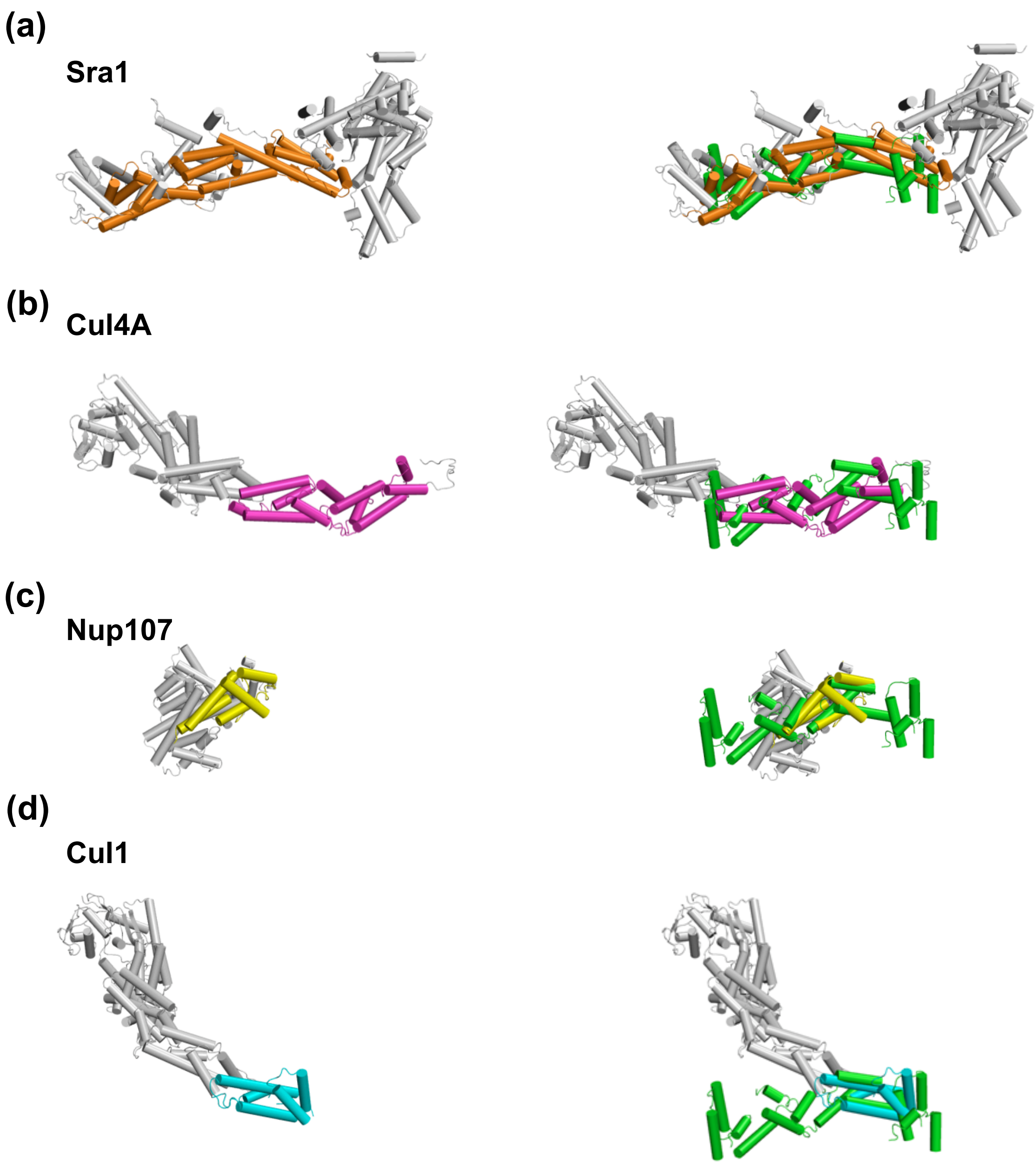

Figure S4. Proteins with high structural homology to CmTic110$_C$ identified by DALI. Structures on the left are structures of the proteins identified by DALI. The colored region is aligned to CmTic110$_C$. Structures on the right are superimpositions with CmTic110$_C$ (green).
(a) Sra1 in the WAVE regulatory complex (3p8c).
(b) Cullin Cul4A in the damage-specific DNA binding protein 1 (DDB1)-Cul4A-Roc1-SV5-V complex (2hye).
(c) Nuclear pore complex protein Nup107 (3i4r).
(d) Cullin Cul1 in the Cul1-Rbx1-Skp1-F box^{Skp2} complex (1ldj).

Figure S5

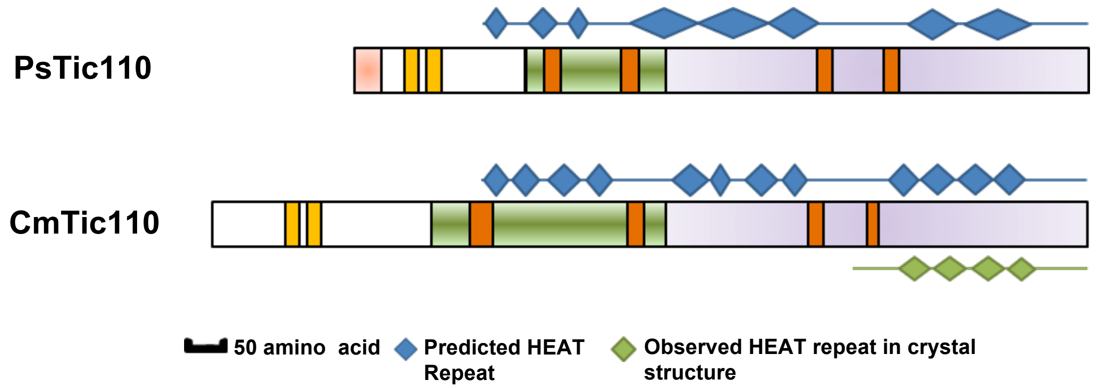

Figure S5. HEAT repeat prediction of pea Tic110 (PsTic110) and CmTic110 using HHrepID.
Domain coloring is the same as Figure 1a. The HEAT repeats are presented as blue diamonds above each protein. The four HEAT repeats observed in our structure are presented as green diamonds below CmTic110.
