# Supplementary material for: Structural characterizations of the chloroplast translocon protein Tic110
Source: Plant J. 2013 May 25;75(5):847–57. doi: 10.1111/tpj.12249 (PMC3823011; doi:10.1111/tpj.12249)
Supplement: Supplementary file 2 [file tpj0075-0847-SD2.pdf]

Table S1. Data-collection and scattering-derived parameters of the SAXS analyses

	$CmTic110_B$	$CmTic110_C$
**Data-collection parameters**		
Instrument	NSRRC 23A	NSRRC 23A
Beam geometry	5 mm slit	5 mm slit
Wavelength (Å)	0.885	0.885
q range ($Å^{-1}$)	0.0065-0.3342	0.0138-0.3999
Exposure time (min)	5	5
Concentration (mg ml^{-1})	1	1
Temperature (K)	277	277
**Structural parameters**		
I(0) (cm^{-1}) [from P(r)]	0.431	0.066
Rg (Å) [from P(r)]	71.63	52.84
I(0) (cm^{-1}) (from Guinier)	0.422	0.064
Rg(Å) (from Guinier)	66.5	49.2
Porod volume estimate ($Å^3$)	207,267	95,916
Dry volume calculated from sequence ($Å^3$)	134,559	96,366
**Molecular-mass determination**		
Partial specific volume ($cm^3 g^{-1}$)	0.729	0.729
Contrast (e $Å^{-3}$)	0.436	0.435
Molecular mass Mr [from I(0)]	172,000	79,600
Calculated monomeric Mr from sequence	59,903	42,784
**Software employed**		
Primary data reduction	NSRRC 23A homemade program	
Data processing	ATSAS	ATSAS
Ab *initio* analysis	GASBOR	GASBOR
Validation and averaging	DAMAVER	DAMAVER
Rigid-body modeling	N/A	N/A
Computation of model intensities	-	CRYSOL

Table S2. Sequences of primers used in this study

Primers	Sequences
CmTic110-F1	gctctagaatgcagagtcctgcggaagc
CmTic110-R1	tcccccgggtcaggcggcttggattcccaac
CmTic110A-F1	ggaattccatatgtcgattggtgcaccgcgagcgc
CmTic110B-F1	ggaattccatatggcaaatcgaagcgagtttacggacgc
CmTic110C-F1	ggaattccatatgcggggtcaggatgaagagcgcg
CmTic110-R2	caactcgagggcggcttggattcccaacatctc
CmTic110-D363amber-F	gaaaatttcgtctatattgactagtatattcgcgcacaagacgtc
CmTic110-D363amber-R	gacgtcttgtgcgcgaatatactagtcaatatagacgaaattttc
CmTic110-F5-HindIII	cgaagcttatgcagagtcctgcggaag
CmTic110-R5-XbaI	cgtctagactagtcaatatagacgaaattttc
