# Supplementary material for: Structural characterizations of the chloroplast translocon protein Tic110
Source: Plant J. 2013 May 25;75(5):847–57. doi: 10.1111/tpj.12249 (PMC3823011; doi:10.1111/tpj.12249)
Supplement: Supplementary file 3 [file tpj0075-0847-SD3.doc]

**SUPPLEMENTAL FIGURE LEGENDS**

**Figure S1.** Secondary structure predictions of CmTic110.

Secondary structure prediction was performed using PHYRE2. CmTic110 is predicted to have an all α-helices structure and the predicted α-helical regions are marked as green tubes under the sequence. The HEAT repeat regions predicted by HHrepID are indicated as magenta tubes below the PHYRE2 secondary structure prediction.

**Figure S2.** Putative oligomerization of CmTic110_C_.

(a) CmTic110_C_ extended into a super-helical structure in crystal packing. For clarity, only the Cα backbone of the molecules is shown. Each monomer is colored with green or blue.

(b) The head-to-head and tail-to-tail dimers were fitted with the scattering data with CRYSOL with the χ value shown. The structures are shown in cartoon with A subunit in green and B subunit in cyan.

(c) Combining the SAXS envelopes of CmTic110_B_ (cyan) and CmTic110_C_ (green) and the crystal structure of the tail-to-tail dimer of CmTic110_C_.

**Figure S3.** Dynamic light scattering measurements of CmTic110_B_ and CmTic110_C_**.**

(a) and (b) Left panels show the intensity autocorrelation function of CmTic110_B_ and CmTic110_C_, respectively, measured by dynamic light scattering (filled circles) and the data fitting by the cumulant expansion (open circles). Size distributions of particles extracted from the autocorrelation function of both proteins are shown in the right panels. The calculated Stokes radius is 73 Å for CmTic110_B_ and 50 Å for CmTic110_C_.

**Figure S4.** Proteins with high structural homology to CmTic110_C_ identified by DALI. Structures on the left are structures of the proteins identified by DALI. The colored region is aligned to CmTic110_C_. Structures on the right are superimpositions with CmTic110_C_ (green).

(a) Sra1 in the WAVE regulatory complex (3p8c).

(b) Cullin Cul4A in the damage-specific DNA binding protein 1 (DDB1)-Cul4A-Roc1-SV5-V complex (2hye).

(c) Nuclear pore complex protein Nup107 (3i4r).

(d) Cullin Cul1 in the Cul1-Rbx1-Skp1-F box^Skp2^ complex (1ldj).

**Figure S5.** HEAT repeat prediction of pea Tic110 (PsTic110) and CmTic110 using HHrepID.

Domain coloring is the same as Figure 1a. The HEAT repeats are presented as blue diamonds above each protein. The four HEAT repeats observed in our structure are presented as green diamonds below CmTic110.

**Table S1.** Data-collection and scattering-derived parameters of the SAXS analyses.

**Table S2.** Sequences of primers used in this study.
